# Effect of age and gender on dietary patterns, mindful eating, body image and confidence

**DOI:** 10.1186/s40359-023-01290-4

**Published:** 2023-09-05

**Authors:** Jinsa Sara Jacob, Neeraj Panwar

**Affiliations:** Department of Psychology, Christ (Deemed to be) University, Delhi, NCR India

**Keywords:** Age, Gender, Dietary patterns, Mindful eating, Body image, Confidence

## Abstract

The emergence of ‘Diet Culture’ came into existence with the era of pop culture, which emphasized the idea of body improvement by embracing the portrayal of unrealistic beauty standards set by the thin-ideal media. This growing and trending culture gained its popularity in India with the COVID pandemic and the imposed lockdown, wherein the prevalence of obesity and binge eating resulted from counter-regulatory eating behaviors and restrictive food intake to a greater extent of skipping meals to achieve the desired body type. The present empirical investigation focuses on understanding the gender and age-based differences (between the ages 18 to 55) among Indian population on dietary patterns, body image, mindful eating and physical appearance confidence using 2 × 3 factorial design. The tools used were Eating Behavior Pattern Questionnaire (EBPQ) [43], Body Self- image Questionnaire (BSIQ) [40], Mindful Eating Questionnaire (MEQ) [18] and Personal Evaluation Inventory (PEI) [44] were administered on a sample size of 120, selected using convenience sampling technique. The collected data was analyzed using SPSS Version 20.0. Results of the study reveal non-significant age and gender differences for mindful eating and appearance confidence. Significant age- differences were observed for Snacking and convenience *F(2,114) = 6.22, p < .05*; social dependence *F(2,114) = 3.87, p < .05* and height dissatisfaction *F(2,114) = 8.79, p < .05*. And, significant gender differences were observed for Meal Skipping *F(1,114) = 6.46, P < .05*; snacking and convenience *F(1,114) = 4.19, p < .05*; fatness evaluation *F(1,114) = 5.94, p < .05* and fitness evaluation *F(1,114) = 5.33, p < .05*. The only significant interaction effect observed was for social dependence dimension *F(2, 114) = 3.96, p < .05*. Thus, high exposure to social media and diet-related content contributed significantly to changing dietary patterns, and how they look, feel or perceive their body.

**Supplementary Information:**

The online version contains supplementary material available at 10.1186/s40359-023-01290-4.

## Background

Over the past 100 years, the portrayal of beauty standards has seen a significant change that was brought about by the pop culture era that was popularized by motion pictures, nightclubs, publications, and consumer goods companies that embraced body renovation. By the early 20th century, the depiction of attractive and slender figures in front page magazines (such as CosmoGirl, Teenpeople, etc.) had exacerbated body insecurities, particularly among women who desired a thinner body type and wanted to be physically attractive to meet the unattainable beauty standards [56]. Additionally, the COVID pandemic’s impact and lockdown over the past two years has led to increased screen time, altered food and lifestyle habits [31], and decreased physical activity, causing complaints of weight gain led people to become active on social media platforms where they were exposed to a high volume of fitness tutorials and weight-loss advertisements, which affected their perceptions of their bodies and led them to engage in low-calorie diets, workouts, and to a greater extent, skipping meals, in an effort to achieve the desired appearance [2].

### Gender differences in perceived body image

Due to the promotion of slim female main characters and the portrayal of obese main characters as being bodily unhappy, the concept of “Body Image” has been so prevalent and misinterpreted by millions of viewers since the invention of television. Such portrayal stressed the concept of “body improvement,“ attempting to meet aesthetic standards that go beyond body shape, height, and weight to include fair skin tones, defined food habits, and fashionable apparel to feel ‘confident’ about one’s body image. And those who do not meet these beauty standards are subjected to intense social pressure and fat shamed by family members, friends, and others, conditioning teenagers and young adults to believe in unachievable ideals of beauty [4]. These expectations for beauty led to feelings of guilt, inadequacy, inferiority, self-doubt, and identity crises [25]. Seven in ten women and girls report having low body confidence and appearance anxiety, and nine out of ten women strive for a healthy body by worshiping thinness. Several research studies have highlighted the role thin-ideal media has played in influencing women’s perceptions of their bodies [27, 41], around 59% of female teens are insecure about their body shape. In his study, Shejwal hypothesized that the development of body dissatisfaction among Indian women is related to perceived social demand for thinness, namely because of their ongoing quest for approval from the media, family, and friends [38].

The highly overlooked reality– body dissatisfaction among male population equally under pressure to conform to societal norms, underestimate their muscularity and body weight [16], ends up using steroid or dietary supplements, which further has negative effects on their physical and mental health [34]. Due to the unrealistic beauty standards of today, both men and women undergo various surgical procedures to achieve the “perfect physical appearance,“ and those who cannot afford the discomfort and costs, opt for exercise, fitness, or dietary restrictions to lose weight or to get a slimmer figure.

### Age- specific presentation of dietary/ restrictive eating behaviors

Additionally, prior studies have demonstrated that dieting or a “restrained” eating style is linked to eating disorders, melancholy, low self-esteem, negative body image, and overeating [33]. A spiral link exists between body image, depression, and self-esteem, with self-esteem being a risk factor for body dissatisfaction in adolescent girls and depressive mood being a risk factor for dissatisfaction in adolescent boys [34].

Studies have placed a strong emphasis on the overly concerned population (adolescents and young adults) with aesthetic values and flaws in one’s perceived appearance, which is a potential risk for developing distorted body image and engages in maladaptive behaviors to ameliorate the dissatisfaction progressing to eating disorders. Teenagers are more motivated by diet and health-related media messages that feature celebrity endorsements [26]. However, middle-aged people’s perceptions of their bodies, changes in dietary habits, and food intake have received relatively little research.

### Unbearable weight of diet culture

Dieting was once thought to be the quickest way to get a slender body type, thus the fascination with dieting is not a new phenomenon. Later, in reaction to societal body ideals, such unrealistic norms became social obligations. The diet industry, often known as the “weight loss industry,“ in addition to the beauty industry, encourages slim-fast and makes untrue claims about how to achieve the “correct body size” by promoting weight loss programs and various diet plans with low-fat and calorie supplements [51]. Nowadays, social media platforms like Instagram and Tiktok have brought the rising and popular “diet culture” trend into attention described as a set of beliefs that supports weight loss as a way to achieve higher moral standards and good health [22]. This trend is outpacing calorie-reduced eating habits that promote weight loss in order to obtain a respectable physical appearance. People start dieting believing they are practicing healthy habits, but the reality is that they are actually engaging in unhealthy activities [3], which prevents them from tuning into what their bodies need. As a result, while you are under the control of a diet mentality, you cannot eat intuitively.

Today’s society is obsessed with having the ideal body shape and weight, not for reasons of health improvement but more for the traits that diet culture in our society represents: being attractive, acceptable, and successful [7]. Cross-cultural research showed that Japanese women were less likely than US women to eat in reaction to emotional states and more likely to engage in dietary behaviors based on physical (associating food with pleasure rather than health goals) and environmental reasons [48]. U.S. women changed their eating habits in reaction to watching television or movies and thinking about how important it was to lose weight, which resulted in more frequent use of restrictive diets, eating disorders, and problematic connections with food [21, 36, 48].

### Pandemic- induced lifestyle changes

Following the COVID outbreak and the subsequent lockdown, the prevalence of obesity and binge eating disorders significantly increased [13] as people used the quarantine period to attempt to lose weight in order to achieve their ideal body type. This is because changes in lifestyle, food preferences, and levels of physical activity have all contributed to weight gain. People began practicing intermittent fasting and low-carb diets like the keto diet, which disrupted people’s peace with food, constantly restricted their hunger cues, and put pressure on their bodies to reach their ideal weight, causing intense dissatisfaction and guilt. These behaviors led to an increase in overeating and disordered relationships with food, which in turn affected “mindful eating.“ Importantly, the mindful eating approach contends that when people are able to let go of diet culture assumptions, they are liberated to make decisions in accordance with their own values [8, 53].

Studies have shown that individuals with high levels of mindfulness also exhibited less problematic eating habits [1] and consumed smaller portions of foods high in energy [5]. There is a need for more study, although the evidence thus far points to the effectiveness of mindfulness-based weight reduction therapies in lowering weight and BMI [9, 50].

As mentioned above, many aspects of diet culture have been investigated separately in earlier studies. A more complete picture about how changing dietary habits affect body image beliefs, self-confidence regarding one’s physical appearance, and to a greater extent the impact on mindful eating remain undiscovered. The COVID pandemic has caused a bigger change in eating habits, food preferences, and lifestyle choices. These changes are geared toward achieving an ideal body type through physical fitness, which is more acceptable by cultural standards of beauty. The influence of diet culture, a constantly evolving concept that encompasses dietary patterns, self-confidence in one’s appearance, relationships with food, and body image assessment, were increasingly prominent and visible in during the pandemic period, across age and gender groups are unaddressed. Given the paucity of research in this area, the current study focuses on evaluating the impact of significant age and gender differences among the Indian population on dietary patterns, body image, mindful eating, and confidence. The study aims to address these dimensions of diet culture among different age groups (between 18 and 55), as well as gender.

### Rationale

Maintaining a healthy diet has always been of utmost significance, but throughout time, viewpoints have shifted due to the cultural preconception that one should be healthy in order to achieve the “ideal body type.“ The focus has shifted to being physically alluring, beautiful, handsome, masculine, and thin due to the pervasiveness of social media platforms and the prevalence of weight loss marketing and advertisements. In order to meet these intolerable standards of beauty, people are motivated to exercise, work out, and diet in order to feel satisfied and accepted by culture. Unsettling findings from Dohnt and Tiggemann showed that by the age of six, up to 42% of girls desired a smaller body, and that 43% were dieting to achieve this goal [15].

There is strong evidence linking the portrayal of thin bodies in the media and women’s body image dissatisfaction, according to a meta-analysis of the body image literature conducted by Gabe and Ward [20]. Approximately 57% of experimental studies found this relationship between thin-ideal body images and women’s dissatisfaction with their own bodies [20], males struggle with body image issues just as much as women do since, in general, males are less likely to seek help for these problems, which leaves their self-perceptions, attitudes, and disordered eating unresolved. Numerous studies have shown that teenagers and young adults who are unhappy with their bodies tend to cut back on their food intake and skip meals in an effort to maintain their weight [10, 55].

Research on middle-aged and older adults in India is lacking, particularly regarding how they view their bodies, how confident they are in their physical appearance as they age, and how their relationship with food has evolved. The current study aims to assess the notable age and gender differences among Indian population on grounds of eating habits, self-image, and confidence while keeping in mind the research gap. The study also considers the concept of “mindful eating”- a non-judgmental conscious awareness of food that adopts a healthy weight management. The study also tries to assess the effect of mindful eating among Indians across different age-groups and gender.

### Research Objective

To assess the effect of age and gender on dietary patterns, mindful eating, body image & confidence among the Indian population.

### Hypotheses

1. There would be no significant main effect of age in Indian population on dietary patterns, mindful eating, body image & confidence.
2. There would be no significant main effect of gender in Indian population on dietary patterns, mindful eating, body image & confidence.
3. There would be no significant interaction effect of age and gender in Indian population on dietary patterns, mindful eating, body image & confidence.

## Method

### Sample

Using the sample size calculation [($N=pq/{\sigma }_{p}^{,2}$), where 0.05 is 1.96 times $\sigma,\, {\sigma }_{p}$ is 0.05/1.96= 0.0255] the target population came to be around 380, but given the pandemic situation, physical interaction with the intended sample size was difficult as the samples also includes middle and older adults. Thus, a total sample of 120 participants were selected using convenience sampling method, a non-probability sampling method where the researchers aim to generate a sample that is accessible and convenient to reach. In order to assess the age and gender- based differences, equal representation of male and female participants was taken i.e. n = 20 males and females from three different age- groups ranging from young adults with an age- range of 18–23, middle adults with an age- range of 30–35 and older adults with an age-range of 50–55.

**Table:** 

	Emerging Adults (18–23)	Middle Adults (30–35)	Older Adults (50–55)
Male	20	20	20
Female	20	20	20

The inclusion and exclusion criterion based on which the data was collected are as follows: Participants within the above listed age group, including both male and female participants and who’re currently residing in India. The participants exceeding the age limits or with any physical impairment or psychiatric disorders or difficulty speaking or following the English language are excluded from the study.

### Participants

#### Descriptives

Participants were 120 Indians, 60 females and 60 males (50% each), with age groups ranging from emerging adults were 41 (34.1%), middle adults were 38 (31.6%) and older adults were 41 (34.1%) selected using convenience sampling. Around 80% of the participants belonged to Upper socio- economic status, educated up till graduation (60%) and post-graduation (50%), among 55% of them are unmarried and 58% are married. Around 59% of the participants are doing a full-time job and 40% of them are pursuing their studies. Among the participants, 80% of them are Non-vegetarian and 20% are vegetarian and around 45% of them follow fitness influencers in social media platforms.

#### Reporting effect sizes

The SPSS software has a feature for running estimates of effect size, so partial eta squared (${n}_{p}^{2}$) was calculated for the main and interaction effects (Age*Gender). The interaction effect size estimates for dietary patterns (DV_1_) ranged from 0.01 to 0.06 (for five dimensions, respectively) indicating small to medium effect of IV on DV. Similar estimates of effect size were found for body image (DV_2_), ranging from 0.01 to 0.06 (for nine dimensions, respectively) showing a small to medium effect of IV on DV. The minimal effect size of 0.04 was also discovered for appearance confidence (DV_3_). However, the effect size for mindful eating (DV_4_) was found to be 0.13 indicating somewhat substantial effect size of IV on DV.

### Research Design

The present study adopts a ‘factorial research design’, a flexible and efficient approach to analyzing results wherein the effects of two or more variables are examined at the same time by making groupings of every combination of the variables. Each independent variable is referred to as a factor, which should include at least two factors or more. And, each factor should include at least two levels or more. The study entails two independent variables i.e. Age with three levels and gender with two levels and four dependent variables i.e. Dietary patterns, Mindful eating, Body image and Confidence. Thus, it highlights a two-factor research design which can be labeled as ‘2 × 3 factorial design’, analyzed using ‘two-way analysis of variance (ANOVA)’ to assess the possible main and interaction effects.

In order to proceed with two- way ANOVA, the data set needs to satisfy the prerequisite assumption of normality of distribution, homogeneity of variances, two independent categorical variables, no significant outliers etc. Using SPSS software, normality tests were carried out in the present study, wherein Normal Q-Q plots showed points lie on a straight diagonal line. The assumption of homogeneity of variances was violated, but Kruskal-Wallis H test (non-parametric test) was carried out, thus retained the H_o_ (p > .05).

### Tools/ measures used

#### Demographic schedule

A demographic schedule was prepared to collect the background characteristics of the target population i.e. name, age, gender, current city, occupation, educational qualification, socio-economic status, marital status, accounts on what all social media platforms, screen time, eating habits, and if following any fitness influencers and if they’re currently engaging in any kind of dietary behaviors.

#### Eating behavior pattern questionnaire [EBPQ; Schlundt, Hargreaves & Buchowski, (2003)

The EBPQ consists of 51 self-report items to assess the determinants of healthy and unhealthy eating behavior through six dimensions. First dimension of the questionnaire is *low fat eating* measured through 11 items (3, 4, 6, 7, 12, 19, 23, 30, 40, 46 & 50); second dimension is *snacking and convenience* which has 10 items (11, 13, 14, 22, 31, 41, 42, 43, 44 & 47); *emotional eating* is the third dimension which has 8 items (2, 9, 10, 15, 17, 20, 28 & 33); fourth dimension is *haphazard meal planning* with 6 items (8, 21, 27, 45, 48 & 51); *meal skipping* is the fifth dimension which has 7 items (18, 24, 26, 36, 37, 38 & 49); and final dimension is *lifestyle behavior* evaluated through 9 items (1, 5, 16, 25, 29, 32, 34, 35 & 39).

Considering the poor performance of Food Habit Questionnaire [6, 47], the developers of EBPQ modified and incorporated new dimensions with a high Cronbach’s alpha of 0.80 for the overall EBPQ [43] and a test-retest reliability ranging from 0.67 to 0.90 [42]. Response category for each statement is based on 5-point Likert’s scale ranging from 1 (strongly disagree) to 5 (strongly agree). There is no cumulate score available for the overall measure rather it gives separate score on six different dimensions. However, to obtain the final scores, total obtained on each dimension has to be divided by the number of statements in respective dimension. Interpretation of the obtained score is quite easy in a sense that if any individual gets an average score of 4 or 5 on any of the dimensions, it reflects that individual has characteristics of that specific eating behaviour.

#### Mindful eating questionnaire [MEQ; Framson & colleagues, 2009

Mindful Eating Questionnaire, self-report instrument consisting of 28-tems with five domains of mindful eating: awareness (consisting of seven items), distraction (consisting of three items), disinhibition (consisting of eight items), emotional responses (consisting of four items), and external cues (consisting of six items). The ratings for eating behaviors are assessed on a 4-point Likert scale; where ‘1 depicts never/rarely’, ‘2 depicts sometimes’, ‘3 depicts often’, and ‘4 depicts usually/always’. Each point values are assigned for each item under five dimensions followed by adding those points and dividing it by total no. of questions being answered to obtain the individual dimension score. And, in order to obtain the overall score, the scores of each dimension are added and divided by 5. Higher scores on the mindful eating questionnaire overall, reflects long-term weight maintenance. The validation for this instrument has been proceeded previously among healthy adults with an age range between 18 and 80 years old. Previous literatures have shown moderate internal consistency with a Cronbach alpha of 0.64 for the MEQ score. Moreover, each subscale reflects fair internal consistency ranging from 0.64 to 0.83 [18].

#### Body self-image questionnaire [BSIQ; Rowe, Benson & Baumgartner, 1999

Body Self-Image Questionnaire (BSIQ) aims to measure the body image construct in a more comprehensive and systematic way. The BSIQ consists of 39 items and a Likert-type 5-point response scale (Not at all true of myself = 1, Slightly true of myself = 2, About halfway true of myself = 3, Mostly true of myself = 4, and Completely true of myself = 5) for each item On development of the questionnaire, the results revealed nine subscales, Overall appearance evaluation, fairness evaluation, fitness evaluation, negative affect, social dependence, attention to grooming, height dissatisfaction and health influence. The nine subscales of the BSIQ are added separately. Interpretation of the scores reveal that higher scores reflects an individual’s negative evaluation of their overall appearance and negative feelings towards their bodies as compared to low scores who express less negative feelings towards their health/fitness. The items have an internal consistency ranging from 0.68 to 0.92. The preliminary results also showed that BSIQ is a validated and reliable instrument by offering a multidimensional measure of body image.

#### Personal evaluation inventory [PEI; Shrauger & Schohn, 1995

The Personal Evaluation Inventory (PEI) is a 54-item scale designed to assess self- confidence or one’s capability over a range of situations [44]. The PEI measures six dimensions of self- confidence including academic performance, athletics, physical appearance, romantic relationships, social interactions and speaking with people. Findings reported Cronbach’s alpha coefficients for women and men, wherein for women it ranges from 0.74 to 0.89 and for men it ranges from 0.53 to 0.89 [44]. Moreover, they reported high convergent validity with the Rosenberg Self-esteem scale. The participants were assessed on a 4- point scale from strongly agree to strongly disagree, where 1 = Strongly agree, 2 = Mainly agree, 3 = Mainly disagree and 4 = trongly disagree. Higher scores depict higher self- confidence, yielding a fair estimate of internal consistency for the following eight subscales reflecting high Cronbach alpha i.e. 0.71 for General, 0.86 for Speaking and Romantic, 0.90 for Athletics, 0.82 for Social, 0.83 for Appearance, 0.77 for Academic, and 0.85 for Mood [44].

### Procedure

After receiving the IRB approval and ethical clearance from Research Conduct and Ethics Committee (RCEC) Christ University, the data collection process proceeded, a participant information sheet was sent to the participants via email/whatsapp invite highlighting the partial details of the research, inclusion criteria for participation, the process of collecting data and the potential ethical guidelines followed by a participant consent form. Once the participants agreed to consent, all the four scales- EBPQ, MEQ, BISQ and PEQ along with demographic schedules were sent to the participants via google forms. The participants were given a three- week period to fill in the responses with reminders. And, once the data was collected, responses were scored and further analyzed using two-way analysis of variance (ANOVA) using SPSS Statistics to assess the effect of independent variables (age & gender) on dependent variables (dietary habits, body image, mindful eating & confidence) followed by main and interaction effects.

### Statistical analysis

The collected data was analyzed using IBM Statistical Program Package for Social Sciences (SPSS) software version 20.0. The demographic details were initially coded (i.e. for Gender category, Female is coded as 1 and Male is coded as 2 etc.) followed by calculating the Descriptive statistics for all the categorical variables (like age, gender, SES, Educational qualification, marital status, occupation, Accounts on what all social media platforms, screen time, eating habits, and if following any fitness influencers). As, all the self- report questionnaires are 4- and 5- pointer Likert scale with each pointer indicating the degree of agreement and disagreement, for questionnaire-wise response format, refer Supplementary Materials. Scoring was further carried out by calculating the average of each dimensions. After data cleaning, the dataset was run in SPSS software for investigating main and interaction effects of the independent variables on the dependent variables carried out by Two- way analysis of variance (ANOVA).

### Ethical considerations

1. Voluntary participation was ensured.
2. Anonymity of the participants was ensured. All the participants were informed that all personal details would remain strictly confidential.
3. Informed consent of all participants was taken before collecting the responses.
4. The participant would be informed that they have the right to withdraw from the study at any point of time they feel uncomfortable.
5. The participant will be debriefed about the nature and purpose of the study after the completion of data collection.

## Results

Mean scores and standard deviations for the dimensions of eating pattern behavior, body self-image, mindful eating and physical appearance with respect to gender and age group is presented in Table 1. And, inferential statistics depicting main and interaction effects are presented in Table 2.

**Table 1:** *Descriptive statistics on dimensions of eating behavior pattern and body self- image across gender and age group (N = 120)*

Dimensions		Gender		Age group		
		Female	Male	Emerging Adults	Middle Adults	Older Adults
Low fat eating	Mean (SD)	34.80 (7.45)	35.93 (7.35)	32.05 (6.61)	35.42 (7.22)	38.63 (6.95)
Emotional eating	Mean (SD)	25.60 (5.31)	24.05 (5.08)	25.22 (6.30)	24.00 (5.03)	25.20 (4.18)
Snacking & Convenience	Mean (SD)	25.57 (8.46)	22.75 (7.19)	27.15 (7.58)	24.11 (8.06)	21.22 (7.24)
Haphazard Meal Planning	Mean (SD)	16.75 (2.80)	17.35 (3.42)	16.73 (3.08)	17.26 (3.15)	17.17 (3.21)
Meal Skipping	Mean (SD)	18.78 (5.36)	16.48 (4.54)	18.85 (5.91)	17.32 (4.61)	16.71 (4.44)
Lifestyle behavior	Mean (SD)	25.08 (6.60)	26.72 (5.81)	25.05 (7.60)	25.47 (5.51)	27.15 (5.27)
Investment in Ideals	Mean (SD)	14.10 (2.79)	14.96 (4.06)	15.29 (3.70)	14.52 (3.82)	13.78 (2.81)
Attention to Grooming	Mean (SD)	9.03 (2.04)	9.15 (2.44)	9.05 (2.42)	9.21 (2.53)	9.02 (1.75)
Fatness Evaluation	Mean (SD)	16.07 (7.28)	13.08 (3.71)	16.07 (8.11)	13.74 (5.51)	13.85 (5.96)
Social Dependence	Mean (SD)	7.43 (2.78)	7.30 (2.15)	8.17 (2.72)	6.79 (2.51)	7.10 (1.99)
Negative Affect	Mean (SD)	8.55 (3.85)	7.78 (3.54)	9.29 (4.93)	7.55 (2.89)	7.61 (2.61)
Overall Appearance Evaluation	Mean (SD)	12.53 (3.29)	12.05 (3.28)	12.41 (3.52)	12.74 (3.48)	11.76 (2.82)
Fitness Influence	Mean (SD)	9.82 (2.28)	9.35 (2.55)	9.66 (2.38)	9.61 (2.63)	9.49 (2.30)
Height Dissatisfaction	Mean (SD)	7.53 (3.89)	6.62 (3.35)	8.88 (3.57)	6.45 (3.68)	5.85 (2.99)
Fitness Evaluation	Mean (SD)	16.13 (3.81)	17.97 (4.77)	16.44 (5.02)	17.87 (4.77)	16.90 (3.20)

*Note: N = 120 (n = 60 for each gender, n = 41 for emerging adults, n = 38 for middle adults and n = 41 for older adults)*

**Table 2:** *Summary table for two-way ANOVA on dimensions of body self- image (N = 120)*

Variables	Factors	*SS*	*df*	*MS*	*F*	Sig
Investment to Ideals	Gender	24.26	1	24.26	2.06	0.15
	Age group	48.67	2	24.33	2.07	0.13
	Gender*Age group	48.43	2	24.21	2.06	0.13
	Error	1337.96	114	11.73		
Attention to Grooming	Gender	0.41	1	0.418	0.08	0.77
	Age group	0.71	2	0.35	0.06	0.93
	Gender*Age group	3.22	2	1.61	0.31	0.73
	Error	593.59	114	5.20		
Fatness Evaluation	Gender	251.77	1	251.77	5.94*	0.01
	Age group	131.86	2	65.93	1.55	0.21
	Gender*Age group	93.62	2	46.81	1.10	0.33
	Error	4831.56	114	42.38		
Social Dependence	Gender	0.117	1	0.117	0.02	0.88
	Age group	43.98	2	21.99	3.87*	0.02
	Gender*Age group	44.99	2	22.49	3.96*	0.02
	Error	646.40	114	5.67		
Negative Affect	Gender	15.59	1	15.59	1.16	0.28
	Age group	77.36	2	38.68	2.88	0.06
	Gender*Age group	10.98	2	5.49	0.40	0.66
	Error	1530.35	114	13.42		
Overall Appearance Evaluation	Gender	7.40	1	7.40	0.67	0.41
	Age group	20.60	2	10.30	0.93	0.39
	Gender*Age group	5.24	2	2.62	0.23	0.78
	Error	1251.97	114	10.98		
Fitness Influence	Gender	6.17	1	6.17	1.03	0.31
	Age group	0.709	2	0.35	0.05	0.94
	Gender*Age group	7.98	2	3.99	0.66	0.51
	Error	681.98	114	5.98		
Height Dissatisfaction	Gender	24.05	1	24.05	2.07	0.15
	Age group	203.71	2	101.85	8.79***	0.000
	Gender*Age group	31.20	2	15.60	1.34	0.26
	Error	1320.11	114	11.58		
Fitness Evaluation	Gender	98.37	1	98.37	5.33*	0.02
	Age group	35.82	2	17.91	0.97	0.38
	Gender*Age group	64.08	2	32.04	1.73	0.18
	Error	2103.35	114	18.45		

Note: *Significant at 0.05 p level; **Significant at 0.01 p level; ***Significant at 0.001 p level. SS = Sum of Squares; df = degrees of freedom; MS = Mean of Squares

**Fig. 1:**
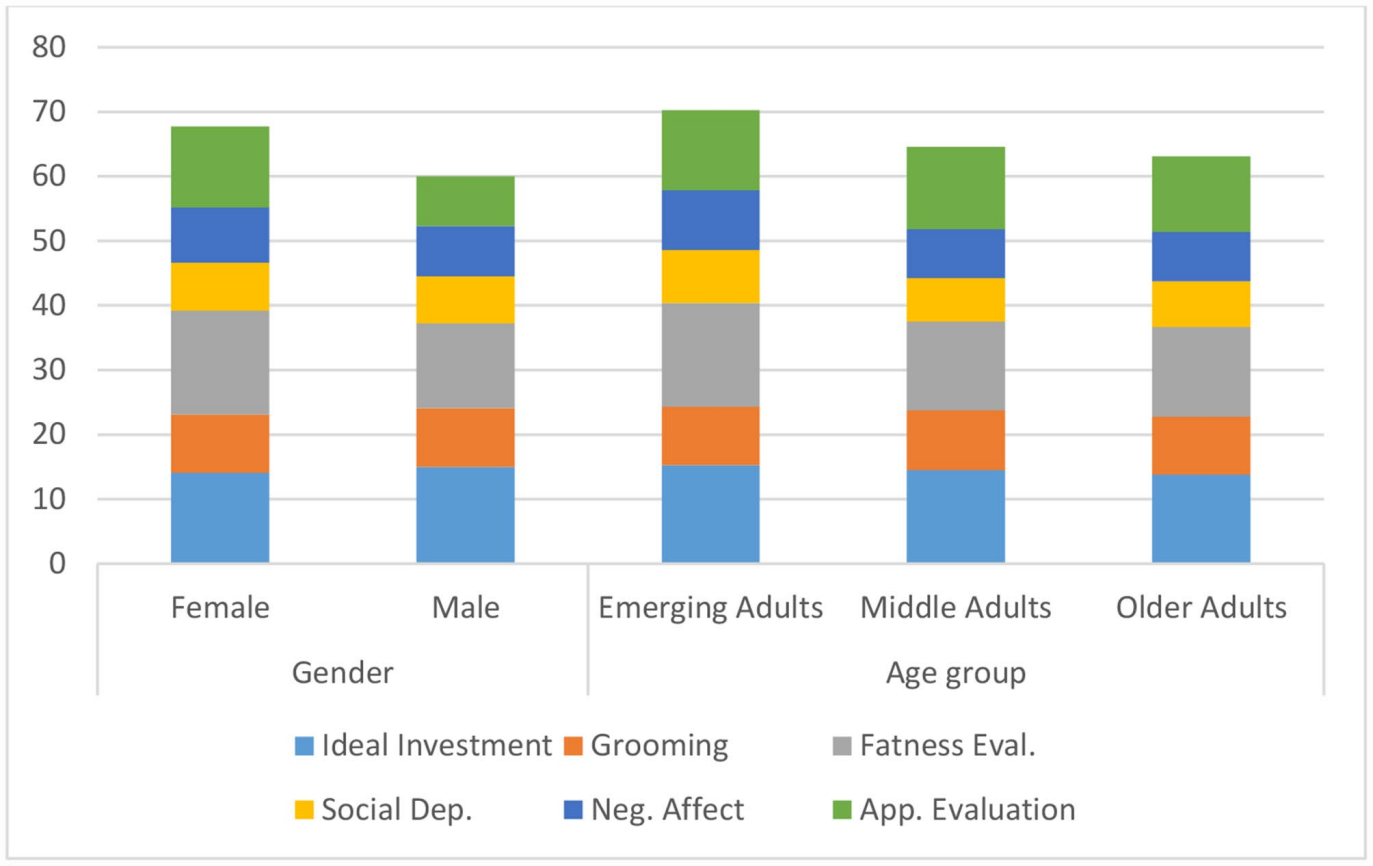
Mean and SD for dimensions of eating behavior pattern for gender and age group

From Table 1, the means of male and female participants with respect to each dimension (Low-fat eating, emotional eating, snacking and convenience, haphazard meal planning, meal skipping and lifestyle behavior) of Eating pattern behavior exhibits a very small difference. The same can also be seen among the three different age groups. Moreover, the highest mean was observed in Older adults for Low-fat eating (*M* = 38.63, *SD* = 6.956) and lifestyle behavior *(M = 27.15, SD = 5.275)* dimensions and lowest mean for Meal skipping *(M = 16.71, SD = 4.440)*, as presented in Fig. 1.

**Fig. 2:**
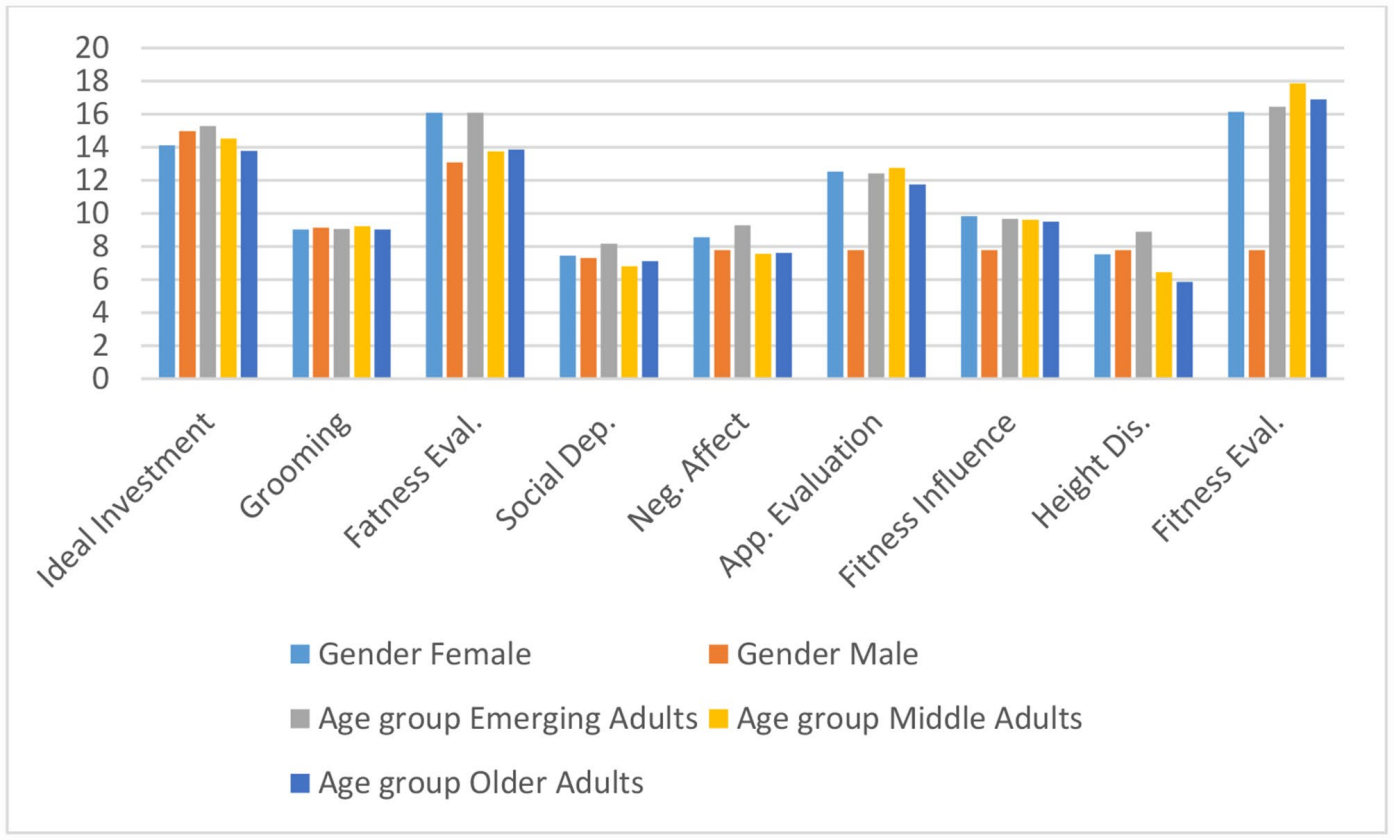
Mean and SD for dimensions of body self- image for gender and age groups

From Table 1, the means of male and female participants for most of the dimension of Body Self- Image exhibits a slight difference. But for fatness evaluation, highest mean was found (*M* = 16.07, *SD* = 7.286). Similarly, for fitness evaluation the highest mean was observed in male middle adults (*M* = 17.87, *SD* = 4.777), depicted in Fig. 2. And, lowest mean was observed in older adults for Height dissatisfaction (*M* = 5.85, *SD* = 2.996) dimension.

From Table 3, significant gender differences was observed for two dimensions i.e. Snacking and Convenience *[F(1,114) = 4.19, p < .05]* and Meal Skipping *[F(1,114) = 6.46, P < .05]*, which indicates that females tend to snack more than males, as presented in Table 1; Fig. 1. Additionally, females are more likely to skip meals than males. A significant difference with respect to the age group was only found for Snacking and Convenience dimension [*F(2,114) = 6.22, p < .05*], wherein emerging adults were seen to snack more than middle and older adults.

**Table 3:** *Summary table for two-way ANOVA on dimensions of eating pattern behavior (N = 120)*

Variables	Factors	*SS*	*df*	*MS*	*F*	Sig
Low- fat Eating	Gender	35.68	1	35.68	0.750	0.38
	Age group	872.72	2	436.36	9.17***	0.001
	Gender*Age group	156.01	2	78.00	1.64	0.19
	Error	5422.26	114	47.56		
Emotional Eating	Gender	71.44	1	71.44	2.64	0.10
	Age group	32.67	2	16.33	0.60	0.54
	Gender*Age group	79.63	2	39.81	1.47	0.23
	Error	3079.36	114	27.01		
Snacking & Convenience	Gender	240.42	1	240.42	4.19*	0.04
	Age group	713.80	2	356.90	6.22**	0.01
	Gender*Age group	26.93	2	13.48	0.23	0.79
	Error	6536.91	114	57.34		
Haphazard Meal Planning	Gender	9.97	1	9.97	0.99	0.32
	Age group	6.332	2	3.16	0.31	0.73
	Gender*Age group	3.88	2	1.94	0.19	0.82
	Error	1146.89	114	10.06		
Meal Skipping	Gender	158.60	1	158.60	6.46*	0.01
	Age group	99.82	2	49.91	2.03	0.13
	Gender*Age group	22.67	2	11.33	0.46	0.63
	Error	2796.37	114	24.53		
Lifestyle behavior	Gender	80.47	1	80.47	2.05	0.15
	Age group	101.92	2	50.96	1.30	0.27
	Gender*Age group	5.86	2	2.93	0.07	0.92
	Error	4460.44	114	39.12		

Note: *Significant at 0.05 p level; **Significant at 0.01 p level; ***Significant at 0.001 p level. SS = Sum of Squares; df = degrees of freedom; MS = Mean of Squares

From Table 2, Significant gender- differences was observed for two dimensions i.e. Fatness Evaluation *[F(1,114) = 5.94, p < .05]* and Fitness Evaluation *[F(1,114) = 5.33, p < .05]*, indicating females to be more concerned about their body weight than males. However, for the fitness evaluation dimension; males showed more concern towards their overall fitness level/ muscle tone than females, as presented in Table 1; Fig. 2. Significant age differences were observed for two dimensions- Social Dependence *[F(2,114) = 3.87, p < .05]* and Height Dissatisfaction *[F(2,114) = 8.79, p < .05]*, wherein emerging adults were more likely to rely on social approval than other age-groups. Similarly, with respect to height dissatisfaction dimension, emerging adults showed more concern and dissatisfaction towards their height wanting to be taller than other age-groups. Additionally, there is a significant interaction between gender and age group with regard to social dependence *[F(2, 114) = 3.96, p.05]*, showing that female emerging adults are more likely than male middle-aged and older adults to depend on social approval for their body image (see Table 1).

**Fig. 3:**
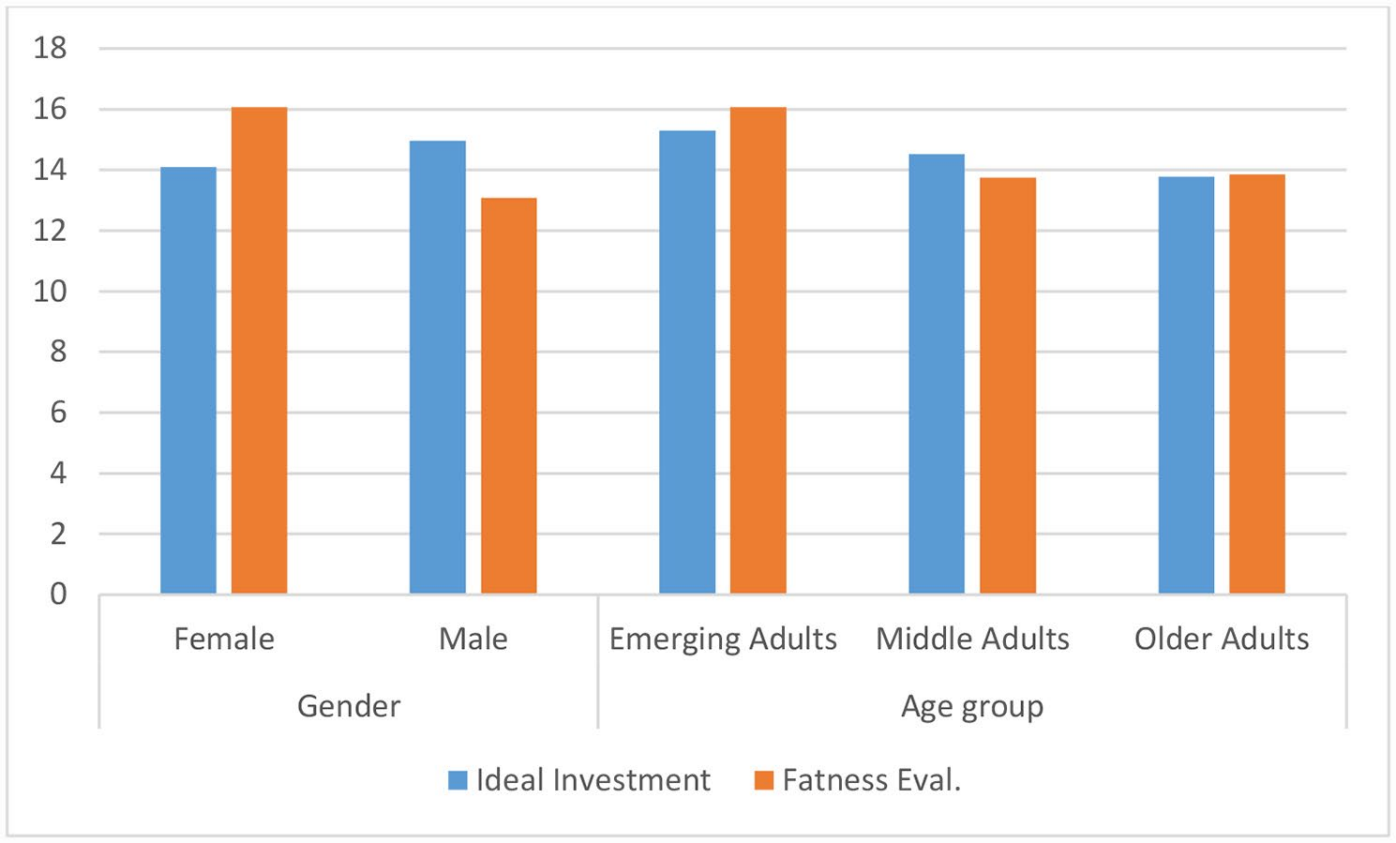
Mean and SD for Mindful eating and Physical appearance confidence for Gender and Age group

From Table 4, no significant gender and age difference with respect to Mindful eating and physical appearance was found. This indicates that both the genders of all age groups equally respond to their hunger or satiety cues and indulge in eating with response to their emotions and equally hold self-evaluative attitudes and feelings towards the way they look and feel about their physical appearance, as depicted in Fig. 3.

**Table 4:** *Summary table for descriptive statistics and two-way ANOVA on Mindful Eating and Personal Evaluation (N = 120)*

Variables	Factors	*M (SD)*		*SS*	*Df*	*MS*	*F*	Sig	
Mindful Eating	Gender	Female	14.12 (1.55)	0.05	1	0.05	0.02		0.86
		Male	14.20 (1.23)						
	Age group	Emerging Adults	13.98 (1.76)	6.06	2	3.03	1.53		0.22
		Middle Adults	14.53 (1.05)						
		Older Adults	14.17 (1.22)						
	Gender*Age group			0.79	2	0.39	0.20		0.81
	Error			225.39	114	1.97			
Personal Evaluation	Gender	Female	17.43 (2.12)	4.45	1	4.45	0.24		
		Male	17.07 (1.42)						
	Age group	Emerging Adults	16.93 (2.18)	8.69	2	4.34	0.27		
		Middle Adults	17.58 (1.70)						
		Older Adults	17.27 (1.45)						
	Gender*Age group			3.13	2	1.56	0.62		
	Error			374.56	114	3.28			

Note: *Significant at 0.05 p level; **Significant at 0.01 p level; ***Significant at 0.001 p level. SS = Sum of Squares; df = degrees of freedom; MS = Mean of Squares; SD = Standard Deviation

## Discussion

The objective of the study is to assess the effect of age and gender on dietary patterns, body image, mindful eating and physical appearance confidence. The experimental study design used four self- report questionnaires: EBPQ, BSIQ, MEQ and PEI on the selected sample size of *N =* 120 using Convenience sampling technique. The current study hypothesized that there would be no significant main and interaction effect of age and gender on dietary patterns, body image, mindful eating and physical appearance confidence.

The results of the present study revealed that there is no significant gender difference on four dimensions of eating behavior pattern i.e. low-fat eating, emotional eating, haphazard meal planning and lifestyle behavior. This is in contrast to the previous finding by Cronin and his colleagues, who found that women were more likely to consume low-calorie food, fruits and carbonated drinks, whereas men reported frequent consumption of meat, dietary products and carbohydrate- rich foods [12]. Numerous studies indicate that females reported to be more vulnerable to restrained, uncontrolled, and emotional eating behaviors leading to cardio and metabolic risk [14, 49, 52]. However, there is a significant gender difference for two dimension of EBP i.e. Meal Skipping and Snacking and Convenience, in line with the findings of Smith and his colleagues and another study by Vereecken and his colleagues found that girls skip breakfast and other meals more frequently than boys as females were more worried about their body weights than males [35, 45, 54]. The current findings reveal that females tend to snack more than males (See Table 1), but previous studies showed mixed results of snacking behaviors, a cross-sectional study reported that women snack more frequently on sweets, biscuits, nuts, and seeds, whereas men tend to snack on salty snacks, add sugar to beverages, and add salt to dishes [57]. Contrary to the present finding, Oba and his colleagues found that boys reported more frequent snacking and eating out as compared to girls [32].

The only significant age differences observed in eating behavior pattern is for the dimension Snacking and Convenience, supporting the findings of Jayawardena, who found that increased snacking behaviors are reported in a high percentage of children, adolescents, and college students in developing countries [23, 46].

Next, no significant gender differences were found for seven of the dimensions (Investment to Ideals, Attention to grooming, fitness influence, social dependence, negative affect, overall appearance evaluation and height dissatisfaction) of Body self-image, which were in contrast with previous findings of Quittkat and his colleagues found that women were more dissatisfied with their bodies than men, due to which women places more importance on their appearance than men, and likely to spend more hours per day on their ideal appearance than men [37]. Significant gender differences were observed in two dimensions (fatness evaluation and fitness evaluation) of body self- image, which is in line with the previous findings of McCreary and Sadava found that women were more conscious about their body than men, women saw themselves heavier than they were, whereas men are more concerned about their overall fitness level and muscle strength than women [28].

The significant age differences were observed for two dimensions (Social dependence and Height dissatisfaction) of body self-image, supporting the findings of [11, 30], stated that older adults tend to experience less body dissatisfaction, and are less likely to indulge in social approval than younger adults. The current finding also revealed a significant interaction effect with respect to the social dependence dimension, which is in line with the previous finding by Gosselink and colleagues found that older adult women, expressed feeling relieved of adherence to societal appearance standards, whereas younger female adults endorse thin-ideal internalization and engage in social comparison contributing to body dissatisfaction [19].

For the mindful eating variable, the results showed no significant age and gender differences which is in contrast to previous findings of Framson, who found that compared to men, women are more aware of their hunger and satiety cues, less distracted while eating and were more mindful. And, in terms of age-differences, he found that as age increased the mindful eating score is higher more towards the middle and older adults [18].

In terms of physical appearance confidence, the result reveals no significant gender and age differences, which is in contrast with the previous findings of Mendelson and his colleagues reported that women who believed that their appearance and body shape were in accordance with the standards set up by the society, reported high level of appearance confidence in contrast to those women holding opposite views [29].

The possible explanation for such insignificant results could be the selected sample for the study, who were highly exposed to social media platforms and the content related to the growing and trending diet culture. As the data collection process proceeded post- pandemic period- a predictive factor contributing to significant changes in people’s eating or dietary patterns, their relationship with food, how they look, feel or perceive their body. Studies have suggested that the pandemic period has contributed towards increased screen time, decreased physical activity, increased snacking behaviors resulting in complaints of weight gain, binge eating and obesity [39]. As a result, individuals started engaging in weight management, highly promoted by the weight-loss industry emphasizing the idea of ‘body improvement’ through their fitness tutorials weight-loss advertisements on low- calorie diets, intense workouts and intermittent fasting to achieve desired appearance and body confidence. People begin to diet with a belief of engaging in health promoting behaviors but the underlying reality is a health compromising behavior, blocking one’s ability to tune into what your body needs, and so; under the thumb of a diet mentality, one cannot eat intuitively [24].

And, these changes in weight-related behaviors can be observed equally in both genders across the age-groups in the present study, which also coincide with the demographic descriptives which reveals that around 47.5% of the sample spend 3–4 h in social media platforms and around 45% of them follows fitness influencers on Instagram. Another possible explanation for such results could be the study population or age- distribution which is greatly varied, might affect the generalizability of the results, which could be one of the potential limitations of the present study. Secondly, the mode of data collection i.e. all the questionnaires are self- report measures which will be sent via google forms, there are chances that the participant might respond in a socially desirable way or being biased in selecting the responses or appearing to be better (faking good) or worse (faking bad) than actual scenario. The lengthy nature of the questionnaires i.e. four self- report measures along with the demographic schedule, was time-consuming and may result in participants engaging in random responding or guesswork. Thirdly, the sampling method which is a non- probability sampling method- Convenient sampling wherein one cannot know how representative the sample is of its intended population. Even the questionnaires used for all variables were not Indianionized scales, which might have affected the responses.

Future researchers should take into account all these limitations and should further explore the influence of other demographic variables on changing dietary patterns, mindful eating and appearance confidence. As, the present study revealed insignificant age and gender differences on mindful eating and physical appearance confidence; thus, increasing the sample size could improve the generalizability. Previous research clearly contradicts the current findings, which revealed that females are more concerned than males about eating behavior, body weight, and physical appearance and have lower self-esteem [17, 35]. Future research should combat the limitations of the current research and can attempt to explore the influence of other demographic variables such as BMI, eating habits, level of physical activity adhering to diet culture by taking into account socio-cultural factors influencing eating patterns, body image perceptions, appearance confidence and mindful eating using a mixed method approach. Future studies on age and gender differences on eating patterns, body image and mindful eating will be important to understand the etiology of eating and body-weight disorders and for designing gender and age-appropriate intervention/ treatments.

## Conclusion

Maintaining a healthy diet has always been a prime importance but with time, the perspectives have changed under the societal preconception of being healthy under the weight of attaining a ‘perfect body’. Due to the pervasive nature of social media platforms, ‘dieting’ became a social obligation that outweighs healthy eating patterns, affecting their relationship/ peace with food in order to feel confident about one’s own body. Results of the present study reveal that there is no significant gender and age-differences on mindful eating and physical appearance confidence. Significant gender- differences were observed for meal skipping, snacking behaviors, fitness and fatness evaluation; and age- based differences were seen for social dependence, height dissatisfaction and snacking behaviors. The only significant interaction effect was observed for social dependence, the potential reason for such significant results in dietary patterns and body image could be due to the pandemic period, causing sudden lifestyle changes (such as maladaptive eating habits, problematic relationship with food etc.) and with increased consumption of diet- content streaming on social media platforms, resulting in internalization of stigmatized media messages on weight gain propagating weight loss- programs, causing negative effects on how we construe or perceive our body image.

### Electronic supplementary material

Below is the link to the electronic supplementary material.

Supplementary Material 1

## Data Availability

Raw data supporting the findings of the study can be obtained from the first author upon request.
