# Supplementary material for: Effect of age and gender on dietary patterns, mindful eating, body image and confidence
Source: BMC Psychol. 2023 Sep 5;11:264. doi: 10.1186/s40359-023-01290-4 (PMC10478183; doi:10.1186/s40359-023-01290-4)
Supplement: Supplementary file 1 — Supplementary Material 1 [file 40359_2023_1290_MOESM1_ESM.docx]

**Appendices**

**Participant Information Sheet**

Hello. I’m Jinsa Jacob currently pursuing Masters in Clinical Psychology from Christ University, Delhi NCR. As a part of the academic curriculum, I am conducting research on understanding health behaviors, one’s relationship with food and how one feels about their body. The detailed information provided below is meant to provide you with an idea of what this research will entail and to seek your cooperation and consent for participation.

**Will I be asked to disclose any personal or private information?**

The participants will be asked to fill in their personal details such as Name, Age, gender, occupation, educational qualifications etc. But we will ensure that all the information shared will be strictly kept confidential. Anonymity will be ensured while analyzing the results.

**Who can participate?**

People fitting the following criteria can participate in this study:

- Age group of 18-23 years, 30-35 years & 50-55 years
- Inclusion of both males and females within the age-range
- Participants should be able to read, speak and understand English

**What will I be asked to do if I agree to participate?**

On agreeing to participate, you‘ll be required to fill up questionnaires which will be sent to you via email or whatsapp and completion of the form will take you around 25 mins. You will be given ample time to fill in your responses followed by few reminders in between to complete the responses within the timeline.

**What if I decide I don’t want to continue the participation?**

If at any point, you feel uncomfortable or want to discontinue your participation, you are free to withdraw after letting us know through a message or email.

**What will this research be used for?**

This research is a part of my academic course. The information and responses that you provide will be documented in my report and further in the later stage will be published as a manuscript (book/document). But all your information will be kept strictly confidential and anonymous.

For further queries, you can get back to me via email: [jinsa.sara@psy.christuniversity.in](mailto:jinsa.sara@psy.christuniversity.in)

**Thank you for your time and interest. I’m grateful!**

**Participant Informed Consent**

I .…………………………………………… hereby consent to participate as requested in the Participant Information Sheet.

1. I have read the information provided
2. I understand what I will be asked to do during the research process
3. I understand that while the information gained in this study will be published as explained, I will not be identified in any way and all of my individual information will remain confidential.
4. I understand that I am free to discontinue or withdraw at any point from the research without any disadvantage

Participant ‘s signature………………………… Date…………………..…

**Instruments**

***Eating Behavior Pattern Questionnaire (EBPQ)***

Mark the box that best describes your level of agreement with each statement.

1= Strongly Disagree, 2= Disagree, 3= Neutral or N.A., 4= Agree, 5= Strongly Agree

1. I stop for a fast food breakfast on the way to work.
2. My emotions affect what and how much I eat.
3. I use low fat food products
4. I carefully watch the portion sizes of my foods.
5. I buy snacks from vending machines.
6. I choose healthy foods to prevent heart disease.
7. I eat meatless meals from time to time because I think that is healthier for me.
8. I take time to plan meals for the coming week.
9. When I buy snack foods, I eat until I have finished the whole package.
10. I eat for comfort.
11. I am a snacker.
12. I count grams.
13. I eat cookies, candy bars, or ice cream in place of dinner.
14. When I don’t plan meals, I eat fast food.
15. I eat when I am upset.
16. I buy meat very time I go to the grocery store.
17. I snack more at night.
18. I rarely eat breakfast.
19. I try to limit the intake of red meat (chicken).
20. When I am in a bad mood, I eat whatever I feel like eating.
21. I never know what I am going to eat for supper (dinner) when I get up in the morning.
22. I snack two to three times a day.
23. Fish and poultry are the only meals I eat.
24. When I am upset, I tend to stop eating.
25. I like to eat vegetables seasoned with fatty meat.
26. If I eat a larger than usual lunch, I will skip supper (dinner).
27. I take a shopping list to the grocery store.
28. If I am bored, I will snack more.
29. I eat at church socials.
30. I am very conscious of how much fat is in the food I eat.
31. I usually keep cookies in the house.
32. I have a serving of meat at every meal.
33. I associate success with food.
34. A complete meal includes meat, a starch, a vegetable, and bread.
35. On Sunday I eat a large meal with my family.
36. Instead of planning meals, I will replace supper with a snack.
37. If I eat a larger than usual lunch, I will replace supper with a snack.
38. If I am busy, I will eat a snack instead of lunch.
39. Sometimes I eat dessert more than once a day.
40. I reduce fat in recipes by substituting ingredients and cutting portions.
41. I have a sweet tooth.
42. I sometimes snack when I am not hungry.
43. I eat out because it is more convenient than eating at home.
44. I hate to cook.
45. I would rather buy take-out food and bring it home than cook.
46. I have at least three to four servings of vegetables per day
47. To me, cookies are an ideal snack food.
48. My eating habits are very routine.
49. If I do not feel hungry, I will skip a meal even if it is time to eat.
50. When choosing fast food, I pick a place that offers healthy foods.
51. I eat at a fast restaurant at least three times a week.

***Body Self- Image Questionnaire***

*Response Format-* Not at all True of Myself**(a),** Slightly True of Myself **(b),** About Halfway True of Myself **©,** Mostly True of Myself **(d),** Completely True of Myself **(e)**Controlling my level of body fat is important to me.

1. I’ve often wanted to be taller.
2. My overall fitness level is high.
3. My thoughts about my body depend on the clothes I’m wearing.
4. My naked body makes me feel sad.
5. I pay careful attention to my face and hair, so that I will look good.
6. I think my body looks fat in clothes.
7. I compare my body to people I’m close to (friends, relatives, etc.).
8. Having a well-proportioned body is important to me.
9. My naked body looks O.K.
10. Being around good-looking people makes me feel sad about my body.
11. I’m usually well-dressed.
12. My body is healthy.
13. Parts of my body are fat
14. I’m more aware of my body when I’m in social situation
15. Muscle definition is important to me.
16. I look good in clothes.
17. My body is fat overall.
18. My naked body makes me angry.
19. I spend time making my appearance more attractive.
20. My overall muscle tone is good.
21. I have large buttocks.
22. How well my body is functioning influences the way I feel about my body.
23. I care about how well-shaped my legs are.
24. I wish I were a different height
25. My body looks good.
26. I feel depressed about my body.
27. My body is strong.
28. My body is overweight.
29. The way I feel about my body improves when I exercise regularly.
30. Body size matters to me.
31. My body is sexually appealing.
32. Most days I feel bad about my body.
33. I have an athletic build.
34. My stomach is flabby.
35. My body image is influenced by the state of my health.
36. My body is in shape.
37. If I were a different height, I’d like my body better
38. I wish I were thinner.

***Mindful Eating Questionnaire***

1—never/rarely, 2—sometimes, 3—often, and 4—usually/always.

1. I eat so quickly that I don't taste what I'm eating.
2. When I eat at “all you can eat” buffets, I tend to overeat.
3. At a party where there is a lot of good food, I notice when it makes me want to eat more food than I should.
4. I recognize when food advertisements make me want to eat
5. When a restaurant portion is too large, I stop eating when I'm full.
6. My thoughts tend to wander while I am eating.
7. When I'm eating one of my favorite foods, I don't recognize when I've had enough.
8. I notice when just going into a movie theater makes me want to eat candy or popcorn.
9. If it doesn't cost much more, I get the larger size food or drink regardless of how hungry I feel.
10. I notice when there are subtle flavors in the foods I eat.
11. If there are leftovers that I like, I take a second helping even though I'm full.
12. When eating a pleasant meal, I notice if it makes me feel relaxed.
13. I snack without noticing that I am eating.
14. When I eat a big meal, I notice if it makes me feel heavy or sluggish.
15. I stop eating when I'm full…even when eating something I love.
16. I appreciate the way my food looks on my plate.
17. When I'm feeling stressed at work, I'll go find something to eat
18. If there's good food at a party, I'll continue eating even after I'm full
19. When I'm sad, I eat to feel better.
20. I notice when foods and drinks are too sweet.
21. Before I eat I take a moment to appreciate the colors and smells of my food.
22. I taste every bite of food that I eat.
23. I recognize when I'm eating and not hungry.
24. I notice when I'm eating from a dish of candy just because it's there.
25. When I'm at a restaurant, I can tell when the portion I've been served is too large for me.
26. I notice when the food I eat affects my emotional state.
27. I have trouble not eating ice cream, cookies, or chips if they're around the house.
28. I think about things I need to do while I am eating.

***Personal Evaluation Inventory***

Below are listed a number of statements that reflect common feelings, attitudes and behaviors. Please read each statement carefully and think about whether you agree or disagree that it applies to you.

A= Strongly Agree

B= Mainly Agree

C= Mainly Disagree

D= Strongly Disagree

1. It bothers me that I am not better looking
2. I am pleased with my physical appearance
3. I am better looking than the average person
4. I am fortunate to be as good looking as I am
5. Most people would probably consider me physically unattractive
6. I wish I could change my physical appearance
7. I would a lot more successful in dating if I were better looking

**Debriefing Manuscript**

There is more to this study than what I have told you about so far. Sometimes in psychological research it is necessary to not tell people about the true purpose of the study at the beginning. If we did, it may affect how they respond to the questions asked and the tasks involved, and this would change the results in a way that may make them invalid.

***Purpose of the study***

The following form was conducted to understand the effect of age and gender on the dietary patterns/ habits, body image, mindful eating and physical appearance confidence.

***Study Procedure***

Once you agree to participate, you will be first asked to provide us with demographic details such as name, age, gender, occupation etc. followed by four self- report questionnaires on measuring your dietary/ food habits, your body image perception, your relationship with food and how confident you feel about your body. The questionnaires will take around 20-25 mins to complete.

***Ethical Guidelines***

- Participation is completely voluntary
- You’re free to withdraw or discontinue from the research at any time without any disadvantage
- Your personal information and responses will be kept confidential and your privacy will be protected. The data shared will be used only for educational purposes.
- There are no direct benefits participating in this research. However, the results may contribute towards future research in the area of diet culture.

We hope that you found your experience participating in this study to be interesting.

Thank you so much for your time and efforts!

For further queries or feedback regarding the research, please feel free to contact via email: [jinsa.sara@psy.christuniversity.in](mailto:jinsa.sara@psy.christuniversity.in)
